# Can a brief session of the online coronavirus disease 2019 destigmatization program reduce stigma among survivors? A randomized controlled trial

**DOI:** 10.3389/fpsyt.2023.1234038

**Published:** 2023-08-23

**Authors:** Kamolvisa Techapoonpon, Chayut Wonglertwisawakorn, Nitchawan Kerdchareon, Wisarat Pruttithavorn, Orranee Srikhamdokkhae

**Affiliations:** Department of Psychiatry, Navamindradhiraj University, Bangkok, Thailand

**Keywords:** COVID-19, coronavirus disease 2019, stigma, online intervention, internet-based intervention, mental health, mental hygiene, destigmatization

## Abstract

**Background:**

Stigmatization has taken a heavy toll on the mental health and quality of life of the survivors of coronavirus disease 2019 (COVID-19). To address this issue, we proposed a brief, self-directed, reflective, and practical destigmatization intervention. The current study aimed to investigate the efficacy of the online COVID-19 destigmatization program (OCDP) in mitigating stigma among the survivors of COVID-19.

**Methods:**

This study was conducted on 142 survivors of COVID-19 before their discharge from Vajra Hospital from July 2022 to November 2022. The participants were randomly assigned between the intervention group (*n* = 71), who attended the 40-min OCDP, and the control group (*n* = 71), who received standard mental health care. The primary outcome was the efficacy of OCDP in reducing stigmatization. A COVID-19 stigma questionnaire was administered to assess stigmatization in the intervention and control groups immediately before and after the program during follow-up on days 7, 14, and 28. The secondary outcome was the efficacy of the program in alleviating negative emotions according to the Depression Anxiety Stress Scale 21 questionnaire.

**Results:**

Compared with the control group, the intervention group had a more prominent reduction in the overall stigma score on day 7 (*p* = 0.002) and day 14 (*p* = 0.028). The intervention group had a more evident reduction in enacted stigma (day 7, *p* = 0.04), internalized stigma (day 7, *p* = 0.008; day 14, *p* < 0.028), and perceived external stigma (day 7, *p* = 0.002) than the control group. However, there was no significant difference in terms of disclosure concern between the intervention and control groups. Furthermore, the reduction in depression, anxiety, and stress between the two groups did not significantly differ.

**Conclusion:**

Online COVID-19 destigmatization program provided prior to hospital discharge is an effective tool in reducing stigmatization, particularly within the first 2 weeks after reintegration into society, among the survivors of COVID-19.

## Introduction

Infectious disease-related stigmatization simply refers to a negative social process of labeling and discriminating people who are believed to be a source of disease (1, 2). This type of stigmatization has long been established. The stigma associated with coronavirus disease 2019 (COVID-19) is due to its high mortality and easy transmission via the respiratory route (3). Furthermore, COVID-19 stigmatization is exacerbated by its emergence in the digital era where patients are subject to cyberbullying among other forms of abuse (4). In the COVID-19 pandemic, stigma has been a global concern with a pool prevalence of 35 percent across all population (5). In Thailand, the prevalence of moderate to high public stigma in 2020 was 75.8 percent, in which, public stigma refers to the reaction that the general population has toward people with COVID-19 (6, 7). Hence, the issue of COVID-19 stigmatization required prior attention addressed.

Physical stigma is associated with delayed diagnosis caused by patients hiding their illness, which can lead to a wider spread of infection (5). Mental health and quality of life are affected by COVID-19 stigmatization, leading to anxiety, depression, and increased suicidal rates (8–10). People experiencing stigma present with prejudice, social exclusion, and discrimination both directly and indirectly, thereby resulting in feelings of blame, shame, and worthlessness (11). Populations at the highest risk of stigma include patients with COVID-19 (12, 13). Although these patients have recovered, they are continuously stigmatized by those around them who are still worried of being infected or those who consider them as unhygienic (1, 14). Hence, patients recovering from COVID-19 need help with reintegration into society.

Previously, anti-stigma interventions existed at several levels from public media and community-based programs to family and individual programs. Nevertheless, the most effective type of interventions often occurs at the individual level, specifically brief interventions that can be easily accessed by patients in hospital services (15, 16). Furthermore, the most effective anti-stigma interventions are provided in workshop or counseling formats, which require large numbers of personnel and become practically challenging during periods where social distancing is imperative (15). Alternatively, the efficacy of simple video-based interventions in the absence of guidance is limited due to low engagement levels from patients (17). Hence, our research team has developed a brief online interactive video-based intervention against COVID-19-related stigma that can reach various populations while decreasing physical contact and human resources and retaining the ability to maintain cooperation with program attendees. This program addresses both internal (self-stigma) and external (enacted) stigma (11). External stigma can be reduced by promoting insights regarding the importance of personal hygiene. Internal stigma is managed with a combination of effective approaches, namely, facilitating empowerment, raising self-esteem, asking for help, and improving knowledge and attitude (15, 18, 19). In addition, several emotional first aid techniques were included in this program.

The purpose of this study is to evaluate the efficacy of the online COVID-19 destigmatization program (OCDP) before hospital discharge and reintegration into society among the survivors of COVID-19. Further, it aimed to provide a suitable alternative solution for combating stigma in patients with COVID-19 and the potential for applicability in other conditions.

## Materials and methods

### Participants

The calculated sample size per group using the G^*^ power software version 3.1.9.7 was 64. The input parameters included the level of significance (*α)* at 5%, power of 80%, allocation ratio of 1, and effect size of 0.5 (medium). Then, 10% of the sample size was further added to allow adjustment for dropouts (20). Therefore, the final sample size per group was 71.

The participants were enrolled between July 2022 and November 2022. The inclusion criteria were as follows: (1) individuals with a positive COVID-19 confirmatory test, (2) those who were admitted and discharged based on data from the Vajira Hospital system, and (3) those who were aged at least 18 years old. The exclusion criteria were as follows: (1) individuals not fluent in the Thai language, (2) those who had visual or hearing issues, and (3) those who had any underlying psychiatric illnesses [from patient report or having Patient Health Questionnaire-9 (PHQ-9) score > 9]. The participants can withdraw from the study at any time.

### Instruments

#### Evaluation tools

##### General survey questionnaire

This questionnaire included sex, age, marital status, number of children, educational level, occupation, underlying medical illness, family history of psychiatric illness, type of house, cohabitant, number of household members with concurrent COVID-19 infection during the time of data collection, and frequency of contact with relatives or friends (6, 11, 14).

##### COVID-19-related stigma questionnaire

This questionnaire was translated by our team from the original version of Dar et al. (11) This tool was previously used to investigate stigma among the survivors of COVID-19. The translation process involved obtaining permission to translate, forward translation by two independent translators, reconciliation by three research team members, backward translation, and approval from the original author, pilot test, and validation. This instrument has 15 items, and it uses a four-point Likert scale (0: strongly disagree, 1: disagree, 2: agree, and 3: strongly agree). The questionnaire covers four stigma domains, which are as follows: enacted stigma (ES, three items), disclosure concerns (DCs, two items), internalized stigma (IS, three items), and perceived external stigma (PES, seven items). The average content validity index (CVI) rated by five experts (three psychiatrists and two psychologists) was 0.95. Each item had a CVI of >0.79 (21). In performing confirmatory factor analysis, every item had a factor loading of >0.5. The coefficient of Cronbach’s alpha in 300 participants was 0.91 (22). The test–retest reliability measured in 101 samples at 2-week intervals was 0.743 (*p* < 0.001) (23). Table 1 shows the backward translation version. A higher score refers to a more severe stigmatization.

**Table 1:** COVID-19-related stigma questionnaire.

Enacted stigma
1. I feel upset to see the reactions of people around me who know I had COVID-19.
2. I have stopped socializing with those who have reactions toward me when they know I have COVID-19.
3. I have lost some friends because I had COVID-19 infection.
Disclosure concern
4. I am very careful to tell others that I had COVID-19 infection
5. I worry that those who know I had COVID-19 infection will tell others.
Internalized stigma
6. I feel that I am not as good as others because I had COVID-19 infection.
7. COVID-19 infection makes me feel like a bad person.
8. I feel guilty because I had COVID-19 infection.
Perceived external stigma
9. Most people think COVID-19 patients are disgusting.
10. Most people are afraid of those with COVID-19 infection.
11. Most people with COVID-19 infection will be rejected if other people know about it.
12. People I know may treat COVID-19 patients like an outcast.
13. People I know may feel uncomfortable to be around COVID-19 patients
14. People I know may reject COVID-19 patients
15. People I know do not want COVID-19 patients to be around their children

##### Depression anxiety stress scale-21, Thai version

This is a 21-item questionnaire on a four-point Likert scale measuring three negative emotional states, namely, depression (seven items), anxiety (seven items), and stress (seven items) (24). This questionnaire was translated from a well-establshed instrument in English version (25). This version has an overall Cronbach alpha coefficient of 0.75. The subscale has Cronbach alpha coefficient of 0.82, 0.78, and 0.69 for depression, anxiety, and stress, respectively.

### Intervention instrument

The online COVID-19 destigmatization program (OCDP) is a video-based learning program provided completely online. In this study, the platform used was a mobile application called AchieveNex. The whole program comprised two modules. Each module can be completed within approximately 20 min. The participants can choose to finish one module at a time. Skipping of the video was disabled. The format of the program significantly involved virtual hosting by a host (MC) who provided information, asked thought-challenging questions, and facilitated self-evaluation, which were the prominent methods used to deliver the information. Each question must be answered before the participants could proceed and complete the session. The participants provided answers by typing in the Google Forms™, which appears after each question.

In terms of content, the first module was an introduction to stigma among patients with COVID-19, which included a definition of stigma, its impact on mental health, and patients’ behavior that can minimize stigma. The second module covered mental preparation for battling stigma, which primarily involved emotional first aid techniques. The CVI of each approach was equal to 1. According to the content validation by ethical board review and experts, there is no contraindication to attending OCDP. Table 2 depicts the full details of the program.

**Table 2:** Outline of online COVID-19 destigmatization program.

Approaches		Techniques	Sequence/Details
Module 1:Introduction to stigma in COVID-19 patients	Empowerment Raising self-esteem Knowledge improvement	Thought-challenging questions Self-evaluation Information provision	Greeting Congratulate on recovery Praise about participants’ courage to detect, disclose, and enter treatment Question about the reactions, the participants might receive after discharge (start from family members, friends to neighbors) Show a video clip of COVID-19 patients who experienced negative reactions and discrimination after return to their society Provide information regarding definition, prevalence, and impact of stigma Ask about participants’ readiness to encounter stigma (rate 1–10) Provide multiple choice question about the person who might be least stigmatized based on their hygiene Point out that the participants can somewhat mitigate stigma by providing information regarding disease transmission to others and complying with government hygienic guideline Ask the participant about how long they think the stigma would last. Provide information about how long the stigma usually last Conclude first module
Module 2:Emotional preparation for battling stigma	Maintain social connection Call for help Preparation Empowerment Cognitive restructuring Emotional recognition Distraction Ventilation	Thought-challenging questions Self-evaluation Information provision	Greeting Use open-ended questions asking about the participants’ feelings if they encounter stigma after returning home Introduce emotional self-care; its importance, use and benefit Empower the participants on their own ability to deal with negative emotions Provide information regarding basic negative emotions and the importance of emotional awareness (use of metaphor) Ask the participants to evaluate their real-time emotions Provide information regarding how to challenge or reframe thoughts that may affect their emotions Give scenario of a recovered COVID-19 patient who return home and found that his close neighbor may try to avoid him Ask: what emotion that person might experience and why that is. Ask: what might be the reasons behind the neighbor’s behavior Provide information regarding different thoughts that may result in different emotions Point out that the display of rejection and discrimination from others is toward the disease not the patient themselves Provide information regarding other ways to cope with negative emotions Distraction; definition, benefits and techniques (counting things and focus on breathing) Ventilation; definition and benefits Exercise; benefits Maintain online social connection; benefits Call for help; importance and benefits Ask the participants to list the people who they can ventilate or seek help from Conclude and empower the participants on emotional self-care Ask about participants’ readiness to encounter stigma (rate 1–10)

### Data collection

**Figure 1:**
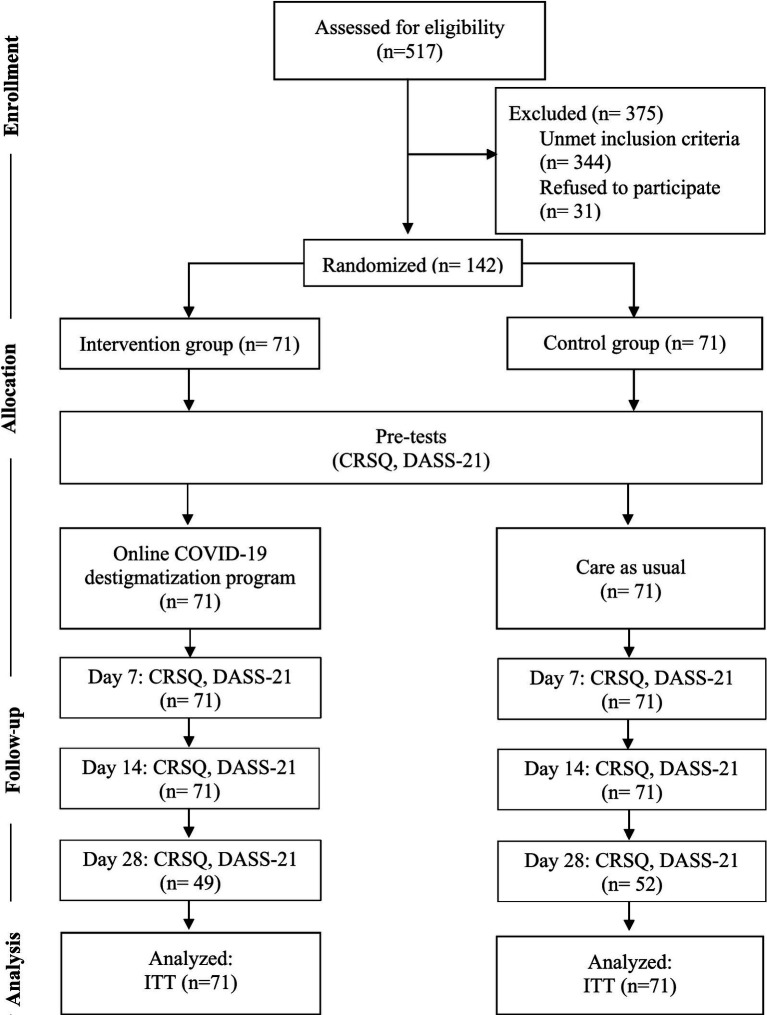
Participant flow.

The current research was approved by the Institutional Review Board of Vajira Hospital (COA 218/2564) and was registered in Thai Clinical Trials Registry (TCTR20220727001). This was a randomized controlled trial conducted from July 2022 to November 2022. All COVID-19 patients in Vajira Hospital received standard mental health care during their admission, which comprised the following: exposing participants to short video clips of relaxation techniques (muscle relaxation, breathing exercise, and self-massaging). Our team contacted the patients who met the inclusion criteria and informed them about the study that could be conducted via a chat application and/or telephone. After excluding patients according to the criteria, the participant information sheet and consent form were provided online using Google Forms™. The participants gave consent by typing their names and checking “I agree to participate” box in the consent forms. After that, the participants were assigned into the intervention (*n* = 71) and control (*n* = 71) groups using simple random sampling method. Both groups were requested to answer the general survey, DASS-21, and CRSQ during the pretests. The intervention group received OCDP approximately 1–2 days prior to discharge. Subsequently, the follow-up tests were performed using DASS-21 and CRSQ on both groups on days 7, 14, and 28 after discharge. On the fourth assessment, some patients were lost to follow-up due to no response in the chat application and telephone. Participants’s data were coded and anonymized in the case record form to protect participants’ confidentiality. Contamination between groups was avoided by not recruiting participants from the same household and restricting new account registration. Figure 1 depicts the full details of participant flow according to the Consolidated Standards of Reporting Trials guidelines.

### Data analysis

Descriptive statistics were used to evaluate demographic and clinical characteristics. Variables were presented as means and standard deviations for continuous data and as frequencies and percentages for categorical data. The chi-square test and independent *t*-test were used to compare data between the intervention and control groups. Data were analyzed according to the intention-to-treat principle. A linear mixed-effects model with an exchange-correlation matrix was used to correct the correlations of repeated measurements to assess the effects of the program on changes in the trial outcomes. Missing data were handled by multiple imputations. Statistical analysis was performed using the Stata version 13.0 software (StataCorp, College Station, TX, United States). A value of *p* of <0.05 was considered statistically significant.

## Results

### Characteristics of the participants

In total, 142 participants were randomized into the intervention (*n* = 71) and control (*n* = 71) groups. Table 3 shows the baseline characteristics of the participants. In terms of general characteristics, there was no significant difference between the control and intervention groups. The majority of participants were women. Their mean age ranged from 31 to 34 years. Most patients were symptomatic. The two groups did not significantly differ in terms of baseline stigma and negative emotions (Table 4). Approximately two-thirds of the participants experienced stigmatization according to the pretest evaluation. The baseline negative emotions ranged from normal to mild.

**Table 3:** Baseline data of the participants.

Data	Intervention group (*n* = 71)		Control group (*n* = 71)		*p* value
	*n*	(%)	*n*	(%)
Sex
Male	14	−19.7	15	−21.1	
Female	57	−80.3	56	−78.9	0.835
Age; year (mean + SD)	31.83 ± 10.41		33.56 ± 11.32		0.344
Marital status
Single	49	−69	47	−66.2	
Married	18	−25.4	19	−26.8	
Divorced/Separated	4	−5.6	5	−7	0.94
Number of children
None	51	−71.8	46	−64.8	
1	8	−11.3	13	−18.3	
2	11	−15.5	10	−14.1	
More than 2	1	−1.4	2	−2.8	0.609
Education
Primary school	2	−2.8	5	−7	
High school	8	−11.3	8	−11.3	
Diploma	6	−8.5	10	−14.1	
Bachelor’s degree	49	−69	34	−47.9	
Master’s degree or higher	6	−8.5	14	−19.7	0.085
Occupation
Unemployed	2	−2.8	0	0	
Students	12	−16.9	18	−25.4	
Civil servant	28	−39.4	21	−29.6	
Company employee	13	−18.3	12	−16.9	
Freelancer	2	−2.8	3	−4.2	
Business owner	1	−1.4	2	−2.8	
University Employee	13	−18.3	15	−21.1	0.555
Income
0–5,000 THB	6	−8.5	9	−12.7	
5,001–10,000 THB	6	−8.5	11	−15.5	
10,001–15,000 THB	13	−18.3	11	−15.5	
15,001–20,000 THB	13	−18.3	12	−16.9	
20,001–25,000 THB	11	−15.5	10	−14.1	
>25,000 THB	22	−31	18	−25.4	0.742
Cohabitant	15	−21.1	8	−11.3	0.143
Live alone	17	−23.9	13	−18.3	
Friends	13	−18.3	11	−15.5	
Partner	26	−36.6	39	−54.9	
Family					
Type of house
Detached/Semi-detached house	13	−18.3	15	−21.1	
Town house/ Town home	9	−12.7	15	−21.1	
Commercial building	5	−7	5	−7	
Flat/Dormitory/Apartment	29	−40.8	25	−35.2	
Condominium	10	−14.1	7	−9.9	
Slum	5	−7	4	−5.6	0.764
Underlying medical illness
Absent	60	−84.5	61	−85.9	
Present	11	−15.5	10	−14.1	0.813
Family history of psychiatric disorder
Absent	67	−94.4	69	−97.2	
Present	4	−5.6	2	−2.8	0.404
Symptoms of COVID-19
Absent	4	−5.6	4	−5.6	
Present	67	−94.4	67	−94.4	1
Family members who were infected with COVID-19 (mean + SD)	1.25 ± 1.29		1.63 ± 1.56		0.116
Frequency of contact with relative or friends during admission
Never	3	−4.2	3	−4.2	
Every 3–4 days	20	−28.2	24	−33.8	
Every 1–2 days	5	−7	5	−7	
Everyday	43	−60.6	39	−54.9	0.906
Depression
Normal	55	−77.5	46	−64.8	
Mild	5	−7	9	−12.7	
Moderate	6	−8.5	9	−12.7	
Severe	1	−1.4	5	−7	
Extremely severe	4	−5.6	2	−2.8	0.208
Anxiety
Normal	43	−60.6	45	−63.4	
Mild	11	−15.5	10	−14.1	
Moderate	6	−8.5	8	−11.3	
Severe	6	−8.5	3	−4.2	
Extremely severe	5	−7	5	−7	0.848
Stress
Normal	59	−83.1	60	−84.5	
Mild	5	−7	6	−8.5	
Moderate	1	−1.4	4	−5.6	
Severe	3	−4.2	0	0	
Extremely severe	3	−4.2	1	−1.4	0.207

^*^p < 0.05, Analyzed by Frequency, Percentile, Mean ± SD, Chi-square, and Independent Sample T-test.

**Table 4:** Score details of stigma and negative emotions measured by CRSQ and DASS-21.

Outcomes	Intervention (*n* = 71)	Control (*n* = 71)	*p* value
	Mean ± SD	Mean ± SD	
Stigma
Total stigma
Baseline	11.45 ± 10.46	9.86 ± 8.18	0.314
7 days	6.83 ± 7.63	8.48 ± 8.40	0.223
14 days	5.86 ± 7.95	7.01 ± 7.99	0.390
28 days	5.39 ± 8.50	5.54 ± 8.05	0.927
Enacted stigma
Baseline	1.51 ± 2.11	1.39 ± 1.95	0.741
7 days	0.70 ± 1.21	1.01 ± 1.61	0.197
14 days	0.55 ± 1.09	0.89 ± 1.55	0.136
28 days	0.69 ± 1.45	0.50 ± 1.20	0.464
Disclosure concern
Baseline	1.28 ± 1.72	1.07 ± 1.38	0.420
7 days	0.92 ± 1.39	0.73 ± 1.24	0.409
14 days	0.62 ± 1.05	0.59 ± 1.01	0.871
28 days	0.65 ± 1.28	0.50 ± 1.08	0.517
Internalized stigma
Baseline	1.54 ± 2.15	1.06 ± 1.48	0.125
7 days	0.77 ± 1.22	0.90 ± 1.44	0.572
14 days	0.62 ± 1.18	0.69 ± 1.24	0.729
28 days	0.55 ± 1.04	0.42 ± 0.94	0.517
Perceived external stigma
Baseline	7.13 ± 6.03	6.34 ± 5.36	0.411
7 days	4.44 ± 5.25	5.83 ± 5.69	0.131
14 days	4.07 ± 5.81	4.85 ± 5.39	0.412
28 days	3.49 ± 5.72	4.12 ± 5.85	0.588
Negative emotions
Depression
Baseline	3.21 ± 4.15	3.18 ± 3.90	0.967
7 days	1.61 ± 2.12	2.38 ± 3.65	0.125
14 days	1.08 ± 2.03	1.97 ± 3.56	0.070
28 days	1.14 ± 2.06	1.42 ± 2.35	0.527
Anxiety
Baseline	3.70 ± 3.97	4.00 ± 3.23	0.627
7 days	2.39 ± 3.27	2.96 ± 3.30	0.308
14 days	1.51 ± 2.56	2.42 ± 3.19	0.061
28 days	1.41 ± 2.40	2.15 ± 3.20	0.190
Stress
Baseline	3.94 ± 4.74	3.99 ± 4.20	0.955
7 days	2.62 ± 3.03	3.28 ± 4.05	0.272
14 days	2.38 ± 3.23	2.41 ± 3.74	0.962
28 days	2.16 ± 3.65	2.25 ± 3.62	0.905

^*^p < 0.05 and ^**^p < 0.001, Analyzed by Mean ± SD and independent sample *t*-test.

### Efficacy of OCDP in reducing stigma

**Figure 2:**
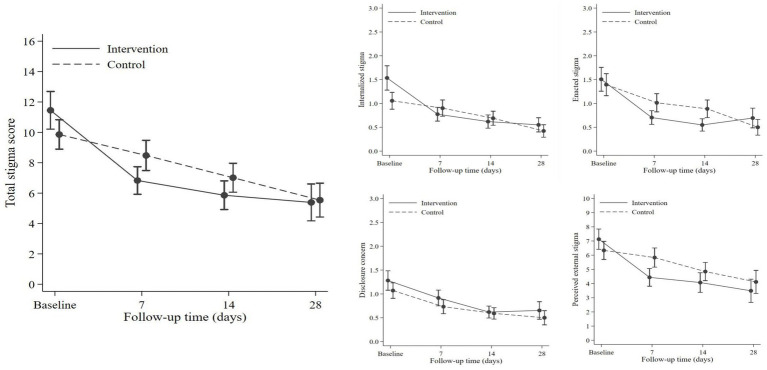
Comparison of total stigma and each domain of stigma between the intervention and control group.

The efficacy of OCDP was evaluated by comparing the mean score and score reduction between the control and intervention groups. There was no significant difference in terms of the mean score between the intervention and control groups (Table 4; Figure 2). However, regarding the score change (Table 5), the intervention group displayed significant reduction since day 7 onwards in both total stigma (*p* ≤ 0.001) and all domains of stigma (ES, IS, and PES; *p* ≤ 0.001, DC; *p* = 0.008). Whereas in the control group, significant score reduction appeared on day 7 only in two domains; ES (*p* = 0.009) and DC (*p* = 0.015). On day 14, the overall stigma score (*p* = 0.001), IS domain score (*p* < 0.001), and PES domain score (*p* < 0.001) of the control group significantly decreased. The intervention group had a significantly more prominent reduction in the overall stigma score on day 7 (*p* = 0.002) and day 14 (*p* = 0.028) than the control group. In each domain of stigma, the difference of score change was observed in ES (day 7; *p* = 0.04), IS (day 7; *p* = 0.008, day 14; *p* = <0.028), and PES (day 7; *p* = 0.002). Nevertheless, there was no significant difference in the DC domain score.

**Table 5:** Comparison of score change from baseline in each visit.

Outcomes	Intervention (*n* = 71)			Control (*n* = 71)			Difference between groups	(95%CI)	*p* value
	Change from baseline	(95% CI)	*p* value	Change from baseline	(95% CI)	*p* value			
Stigma									
Total stigma									
7 days	−4.62	(−6.07, −3.17)	<0.001^**^	−1.38	(−2.83, 0.07)	0.062	−3.24	(−5.29, −1.19)	0.002^*^
14 days	−5.59	(−7.32, −3.86)	<0.001^**^	−2.85	(−4.58, −1.11)	0.001^*^	−2.75	(−5.19, −0.30)	0.028^*^
28 days	−6.11	(−8.90, −3.33)	<0.001^**^	−3.78	(−6.53, −1.04)	0.007^*^	−2.33	(−6.24, 1.58)	0.243
Enacted stigma									
7 days	−0.80	(−1.09, −0.52)	<0.001^**^	−0.38	(−0.67, −0.1)	0.009^*^	−0.42	(−0.83, −0.02)	0.040^*^
14 days	−0.96	(−1.29, −0.63)	<0.001^**^	−0.51	(−0.84, −0.17)	0.003^*^	−0.45	(−0.92, 0.02)	0.060
28 days	−0.84	(−1.36, −0.32)	0.002^*^	−0.88	(−1.39, −0.37)	0.001^*^	0.04	(−0.69, 0.78)	0.906
Disclosure concern									
7 days	−0.37	(−0.64, −0.09)	0.008^*^	−0.34	(−0.61, −0.07)	0.015^*^	−0.03	(−0.41, 0.36)	0.886
14 days	−0.66	(−0.96, −0.36)	<0.001^**^	−0.48	(−0.78, −0.18)	0.002^*^	−0.18	(−0.61, 0.24)	0.397
28 days	−0.71	(−1.14, −0.27)	0.001^*^	−0.54	(−0.97, −0.12)	0.013^*^	−0.16	(−0.77, 0.45)	0.600
Internalized stigma									
7 days	−0.76	(−1.08, −0.44)	<0.001^**^	−0.15	(−0.47, 0.16)	0.340	−0.61	(−1.06, −0.16)	0.008^*^
14 days	−0.92	(−1.26, −0.57)	<0.001^**^	−0.37	(−0.71, −0.02)	0.038^*^	−0.55	(−1.04, −0.06)	0.028^*^
28 days	−1.01	(−1.50, −0.52)	<0.001^**^	−0.51	(−0.99, −0.03)	0.036^*^	−0.50	(−1.18, 0.19)	0.156
Perceived external stigma									
7 days	−2.69	(−3.65, −1.73)	<0.001^**^	−0.51	(−1.47, 0.46)	0.302	−2.18	(−3.55, −0.82)	0.002^*^
14 days	−3.06	(−4.22, −1.90)	<0.001^**^	−1.49	(−2.65, −0.33)	0.012^*^	−1.56	(−3.20, 0.08)	0.062
28 days	−3.63	(−5.51, −1.74)	<0.001^**^	−1.86	(−3.71, 0.00)	0.050	−1.77	(−4.41, 0.88)	0.190
Negative emotions									
Depression									
7 days	−1.61	(−2.23, −0.98)	<0.001^**^	−0.80	(−1.43, −0.18)	0.011^*^	−0.80	(−1.68, 0.08)	0.074
14 days	−2.13	(−2.84, −1.41)	<0.001^**^	−1.21	(−1.92, −0.50)	0.001^*^	−0.92	(−1.92, 0.09)	0.075
28 days	−1.98	(−3.07, −0.89)	<0.001^**^	−1.23	(−2.30, −0.15)	0.025^*^	−0.75	(−2.28, 0.78)	0.337
Anxiety									
7 days	−1.31	(−1.90, −0.72)	<0.001^**^	−1.04	(−1.63, −0.46)	<0.001^**^	−0.27	(−1.10, 0.56)	0.527
14 days	−2.20	(−2.89, −1.50)	<0.001^**^	−1.58	(−2.27, −0.88)	<0.001^**^	−0.62	(−1.60, 0.36)	0.216
28 days	−2.32	(−3.42, −1.21)	<0.001^**^	−1.51	(−2.60, −0.42)	0.006^*^	−0.80	(−2.36, 0.75)	0.310
Stress									
7 days	−1.32	(−2.07, −0.58)	<0.001^**^	−0.70	(−1.45, 0.04)	0.064	−0.62	(−1.67, 0.43)	0.249
14 days	−1.56	(−2.41, −0.72)	<0.001^**^	−1.58	(−2.42, −0.73)	<0.001^**^	0.01	(−1.18, 1.21)	0.982
28 days	−1.84	(−3.13, −0.55)	0.005^*^	−1.46	(−2.73, −0.20)	0.024^*^	−0.37	(−2.18, 1.43)	0.685

^*^p < 0.05 and ^**^p < 0.001, analyzed by linear mixed model.

### Efficacy of OCDP in reducing negative emotions

**Figure 3:**
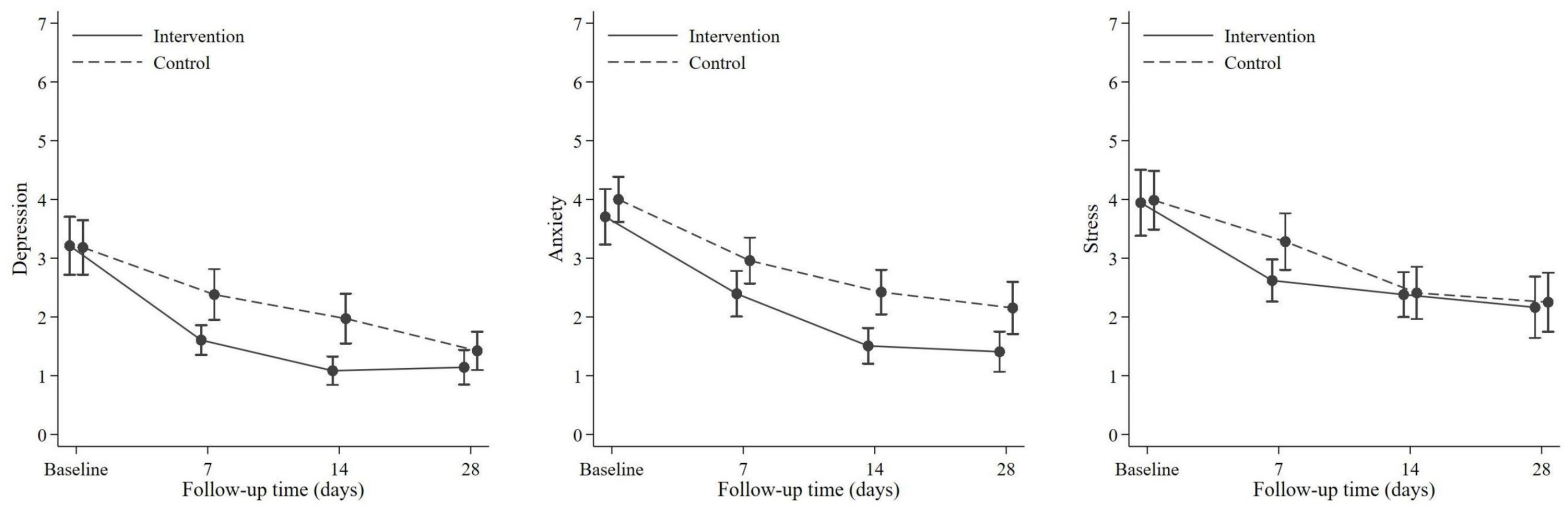
Comparison of depression anxiety and stress between the intervention and control group.

The mean scores of the intervention and control groups were compared (Table 4; Figure 3). Results showed no significant difference of mean score between the two groups. However, as shown in Table 5, the intervention group experienced a significant change in the score for all negative emotions on day 7 (depression, *p* < 0.001; anxiety, *p* < 0.0011; and stress, *p* < 0.001). The control group exhibited a significant reduction in the scores only for depression (*p* = 0.011) and anxiety (*p* < 0.001) on day 7. Stress reduction was significant on day 14 (*p* < 0.001). Nevertheless, there was no significant difference in terms of changes in the score between the control and intervention groups.

### Factors associated with reducing stigma and negative emotions

According to a linear regression analysis, a family history of psychiatric disorder could be a significant predictor of a poor reduction in PES (β = −0.220, *p* = 0.027). No other factors were found to be significant predictors of change in the overall stigma score and other stigma domain score. Regarding negative emotions, the presence of cohabitants could be a significant predictor of anxiety (β = 0.308, *p* = 0.001) and stress level (β = 0.236, *p* = 0.016). *Post hoc* analysis with one-way ANOVA did not show any significant difference among the different types of cohabitants. Further, a family history of psychiatric disorder was a significant predictor of a poor reduction in depression (β = −0.305, *p* = 0.002).

### Additional analysis

We analyzed the response of the intervention group during the program. Based on the independent *t*-test, the participants’ readiness to encounter stigma (score rating: 1–10) improved significantly after receiving the program [from pretest (8.76 ± 1.38) to post-test (9.54 ± 0.88), *p* < 0.001]. The risks of rejection from family members, friends, and neighbors among the participants were 20.6, 38.2, and 57.4%, respectively. According to the participants’ opinion, stigma could last for days (54.4%), weeks (35.3%), months (7.4%), and years (2.9%).

With regard to the question about feelings in case of stigma after returning home, 45.1% of the participants reported feelings of sadness. The answers included “I would feel sad. Nobody wants to contract the disease,” “I would feel depressed and lose my confidence,” “I would feel guilty and disappointed,” “I would feel that nobody wants to come near me,” and “The community could not welcome people who just recovered from COVID-19.” Approximately 8.5% participants had feelings of anxiety. The responses included “I would feel so worried that I would not want to meet anybody” and “Initially, 60% of me feels I would be worried and unconfident to go out, spend time with others, or even walk past other people. And for the rest of 40%, I think I have recovered. I should be able to go out, but I may have to keep my distance. I must wait a while until I can approach other people.” Approximately 5.6% of the participants expressed anger. The rest of the participants in the intervention group stated that they understood the situation and that they were not concerned.

## Discussion

This trial confirmed the efficacy of OCDP in reducing stigmatization among patients with COVID-19 who are reintegrating into society. We hypothesized that its efficacy stems from various elements within the program. First, the intervention is provided at an adequate timing when patients are about to be reintegrated into society. This finding reflects that of previous studies suggesting that destigmatization programs should be implemented early before internalized stigmatization progresses (26). In addition, stigma from COVID-19 infection is highest in the early phases of reintegration into society (27). Regarding the COVID-19 situation during the study period, Thailand exited the state of emergency for COVID-19 in the second half of the trial. However, social distancing, masking, frequent hand hygiene, and self-quarantine were regularly practiced in the society (28). The statistics of COVID-19 patients were as follows: active cases ranged from 4,562 to 23,928 cases per day, new cases ranged from 319 to 2,747 cases per day, and new deaths ranged from 6 to 35 cases per day (29). Second, the content of the intervention is relevant and useful as stigma is extremely common during the COVID-19 and previous infectious outbreaks (16, 30, 31). Our intervention was designed based on the principles of anticipation and acceptance that stigma is likely to occur under normal circumstances (16, 26). Hence, the content was established to prepare patients in managing their mental health in advance. That is, they should have the skills and knowledge in reducing the occurrence and complications of stigmatization (16, 32). Finally, the format of content presentation is based on previous evidence focusing on time efficiency (16). The duration of the whole intervention is 40 min. Further, it is split into two subsections that can be watched separately, which is suitable for the attention span of a typical audience (33). The use of video-based interventions was practical. A previous study has shown that these interventions can be provided with easily and that their efficacy is comparable to that of live interventions (34). The efficacy of video-based interventions is enhanced by message-delivering techniques such as facilitating self-evaluation and asking challenging thoughts, and the use of metaphors, which are important methods in combating the emergence of IS (24). In addition, the opportunity of participants to interact with the intervention material in real time via written responses to questions encourages them to explore their state of mind from their own perspective, thereby enhancing mental preparation for reintegration into society and facing stigma.

The efficacy of our intervention in reducing stigma can be analyzed from several aspects. First, this study showed that OCDP could significantly reduce ES. This result emphasizes the importance of social behavior in individuals infected with COVID-19 and their ability to provide correct information to their cohabitants. These factors influence the resulting attitudes of stigmatization exhibited toward individuals who are infected (1, 14). Second, PES reduction may be attributed to the ability of this intervention to change the perception of participants toward external stigma by normalizing infectious disease-related stigma that can be encountered upon social reintegration, which is often temporary (16, 26, 27). Furthermore, the cognitive restructuring principle was utilized to reduce the misperceptions of behaviors and the reactions of others toward individuals who are infected. IS is the final domain that significantly reduced after OCDP. As previously discussed, the patients could benefit from the effect of early implementation. Further, the program utilized cognitive behavioral therapy for reducing IS, as recommended by previous studies (26). Psychoeducation regarding emotional first aid increases the participant’s resilience in coping with negative emotions, which, in turn, reduces IS (35). Another factor affecting IS includes ES reduction, as a previous study in China found that the COVID-19-related experience of discrimination can positively predict IS (36). Nevertheless, the intervention was limited in its ability to significantly reduce DC in the intervention group. Hence, the intervention could not sufficiently reduce the participants’ fears of being stigmatized if they were found to be infected (37). By contrast, DC may stem from other factors such as the risk of losing income (38). Moreover, it is an important and challenging issue as the willingness to disclose information about infections is important for reducing transmission. Therefore, further interventions are required to address this problematic domain.

The analysis of secondary outcomes in measuring the efficacy of this program found a significant reduction in depression and stress in the intervention group. However, the change did not significantly differ between the intervention and control groups even if stigma reduction was evidently different. This phenomenon could be explained by the followings: First, negative emotions, particularly anxiety, may be associated with DCs, which did not significantly reduce in the intervention group after the intervention (39). Second, it is possible that factors other than stigma may cause negative emotions such as economic causes or mental stress as a result of physical distancing or quarantine (40), therefore these challenging issues require further study.

The implication of OCDP is 2-fold. That is, it can be applied directly during the COVID-19 pandemic or its contents can be modified for patients infected with other diseases. The intervention can be provided easily, is economical and not human resource-intensive, and can be suitable for patients who require social distancing during recovery, such as those with tuberculosis or other respiratory tract infections. We recommend early implementation of the program since it focuses on prevention and the infectious-disease related stigma is highest at the early stage of social reintegration.

### Strengths and limitations of the current study

Online COVID-19 destigmatization program is the first intervention against stigmatization created for patients with COVID-19 who have recovered and are being reintegrated into society in Thailand. This tool contributes to the scientific literature and poses new questions to existing data about destigmatization programs.

The current study had some limitations. First, data collection was conducted in the middle of 2022, which is during the later stage of the pandemic where 80% of the population in Thailand has already received two doses of the vaccines and effective treatments against COVID-19 have already emerged (41, 42). This could have resulted in stigma reduction compared with during the earlier phases of the pandemic (6, 11). Therefore, the efficacy of data on OCDP might not be generalizable to other disease pandemics in different stages of the outbreaks. Second, information regarding vaccination status and number of infection episodes were not collected in this study. Although the majority of the Thai population received full vaccination and breakthrough COVID-19 infection was common in vaccinated people, these factors could play a role in mediating the stigma (41–43). Third, long-term benefit of OCDP was not investigated in this study. Although, the result showed that the effect of OCDP reduced to a statistically non-significant level on day 28, long-term impact is still open to question. Forth, the use of technology is a barrier for accessibility to interventions in specific segments of the society such as the elderly individuals.

## Conclusion

This study showed that providing OCDP prior to hospital discharge is an effective tool in reducing overall stigma, enacted stigma, internalized stigma, and perceived external stigma among the survivors of COVID-19, particularly within the first 2 weeks after reintegration into society. This intervention was designed based on a preventive approach, using the principles of anticipation and acceptance of stigma. The key content includes skills and knowledge in reducing the occurrence and complications of stigmatization. The information was delivered using interactive video presentation, which applicable during social distancing. The results of the present study highlight the importance of prevention and suggest early implementation of the intervention in addressing COVID-19 related stigmatization. Moreover, this program is not only suitable for patients with COVID-19, and its contents and format can also be modified for use in other disease pandemics. However, there are limitations regarding the efficacy of OCDP in reducing disclosure concern and negative emotions, particularly depression, anxiety, and stress.

## Data availability statement

The original contributions presented in the study are included in the article/supplementary material, further inquiries can be directed to the corresponding author.

## Ethics statement

The studies involving human participants were reviewed and approved by Vajira Institutional Review Board, faculty of medicine, Vajira Hospital, Navamindradhiraj University (COA 218/2564). The patients/participants provided their written informed consent to participate in this study.

## Author contributions

KT: conceptualization, designing intervention, methodology, analysis, project administration, and manuscript writing. CW: designing and constructing intervention and submitting manuscript. NK: supervision and funding acquisition. WP: designing and constructing intervention. OS: investigation, statistical analysis, and data curation. All authors contributed to the article and approved the submitted version.

## Funding

This research received funding from the National Research Council of Thailand (N35A650057).

## Conflict of interest

The authors declare that the research was conducted in the absence of any commercial or financial relationships that could be construed as a potential conflict of interest.

## Publisher’s note

All claims expressed in this article are solely those of the authors and do not necessarily represent those of their affiliated organizations, or those of the publisher, the editors and the reviewers. Any product that may be evaluated in this article, or claim that may be made by its manufacturer, is not guaranteed or endorsed by the publisher.
